# Reverse shoulder arthroplasty with an inverted-bearing prosthesis as revision procedure for failed anatomic and reverse shoulder arthroplasty: a long-term multicenter study

**DOI:** 10.1016/j.jseint.2024.05.006

**Published:** 2024-05-28

**Authors:** Falk Reuther, Ulrich Irlenbusch, Georges Kohut, Thierry Joudet, Max J. Kääb

**Affiliations:** a DRK Kliniken Berlin Köpenick, Clinic for Trauma Surgery and Orthopaedics, Berlin, Germany; b Sports Clinic Erfurt, Erfurt, Germany; c Clinique Générale Ste-Anne, Orthopaedics and Traumatology, Fribourg, Switzerland; d Clinique Chirurgicale du Libournais, Orthopaedic Surgery Center, Libourne, France; e Sporthopaedicum Straubing, Shoulder Center, Straubing, Germany

**Keywords:** Revision RTSA, Reverse total shoulder arthroplasty, Anatomic shoulder arthroplasty, Clinical outcomes, Long-term prosthesis survival, Re-revision, Complications

## Abstract

**Background:**

Despite the increasing use of revision reverse total shoulder arthroplasty (RTSA), studies directly comparing revision RTSA performed for different failed index procedures are limited. We therefore compared the results of revision RTSA between patients with a failed primary anatomic arthroplasty (total shoulder arthroplasty and hemiarthroplasty) and those with a failed primary RTSA to explore revision of which index procedure resulted in better long-term clinical outcomes.

**Methods:**

In this prospective, multicenter, observational study, patients underwent revision RTSA using an inverted-bearing prosthesis. We recorded clinical scores, active range of motion, pain, satisfaction, and the rate of scapular notching. Complications and prosthesis survival were also noted.

**Results:**

We included 45 patients (45 shoulders) with revision RTSA for failed primary anatomic shoulder arthroplasty (30 patients) and RTSA (15 patients). Clinical and radiographic outcomes were recorded from 36 patients at a median follow-up of 101.6 months, and prosthesis survival was assessed from all 45 patients. At final follow-up, clinical scores (*P* < .05), abduction (*P* = .032), re-revision rate (*P* = .018), and prosthesis survival (*P* = .015) were significantly better in patients revised from failed primary anatomic shoulder arthroplasty than those from RTSA. However, pain, satisfaction, and overall complication rates were similar in both groups (*P* > .05).

**Conclusions:**

We found better long-term clinical scores, abduction, and prosthesis survival rates after failed primary anatomic shoulder arthroplasty than after RTSA. Pain reduction and complication rates were comparable in both groups. Thus, anatomic shoulder arthroplasty remains an attractive option for primary arthroplasty in selected cases.

Primary anatomic shoulder arthroplasty and reverse total shoulder arthroplasty (RTSA) are both considered standard of care for various shoulder pathologies including proximal humeral fractures, osteoarthritis, and cuff tear arthropathy.^8^^,^^19^ Nevertheless, the use of RTSA as a primary procedure has increased over time due to broader indications and promising clinical outcomes.^7^^,^^25^^,^^31^^,^^35^ According to a recent nationwide analysis in the United States, RTSA comprised 55% of all shoulder arthroplasties in 2016 and increased to nearly 70% in 2020.^25^ In Australia, RTSA has also become the most common type of shoulder arthroplasty, accounting for 66.9% of all total shoulder arthroplasties (TSAs).^13^ And in New Zealand, the incidence of RTSA increased from 2.9 to 16.6 cases per 100,000 people per year from 2009 to 2019.^37^

Similarly, revision RTSA is becoming increasingly popular for revising a failed primary shoulder arthroplasty, mainly because an insufﬁcient rotator cuff is largely responsible for pain, pseudoparalysis, or instability after primary shoulder arthroplasty.^11^^,^^17^^,^^26^^,^^35^ Additionally, revision RTSA has shown to reliably improve function and reduce pain after failed shoulder arthroplasty.^1^^,^^4^^,^^5^^,^^7^^,^^11^^,^^15^, ^16^, ^17^^,^^26^, ^27^, ^28^^,^^33^^,^^35^^,^^36^ However, studies directly comparing the clinical outcomes of revision RTSA performed for failed anatomic shoulder arthroplasty with those of failed RTSA are limited.^14^ As the need for revision shoulder arthroplasty in an aging population increases and the indications of anatomic shoulder arthroplasty and RTSA begin to overlap, clinical outcomes after revision shoulder arthroplasty are more likely to influence the choice of primary procedure, especially in younger patients.

The goal of our study was to explore which failed index procedure resulted in better long-term clinical outcomes once revised, comparing the outcomes of primary anatomic arthroplasty (TSA and hemiarthroplasty [HA]) with those of primary RTSA. Therefore, we compared the results of revision RTSA between patients with a failed primary anatomic arthroplasty and those with a failed primary RTSA for various shoulder pathologies using an inverted-bearing prosthesis system. Additionally, we evaluated prosthesis survival and complication rates in both groups. We hypothesized that revision RTSA performed for a failed anatomic shoulder arthroplasty and a failed RTSA would lead to similar long-term clinical outcomes and prosthesis survival rates.

## Materials and methods

This prospective, multicenter, observational study included some patients from a previously published study.^21^ We prospectively enrolled consecutive patients who underwent revision RTSA between November 2007 and September 2015 from five specialized shoulder centers (three in Germany, one in France, and one in Switzerland). Patients were examined clinically before surgery and at 3, 6, 12, 24, 48, and approximately 84 months for the final follow-up. For survival analysis, patients underwent regular checkups every 2 years until 14 years postoperatively. All patients gave written informed consent to participate in the study and agreed to the follow-up examination.

We operated on patients in a beach chair position under general anesthesia and used a deltopectoral approach in 87% and a deltoid split approach in 13% of shoulders. All patients were treated with the Affinis Inverse Reversed Shoulder Prosthesis System (Mathys Ltd., Bettlach, Switzerland), which has an inverted bearing couple consisting of a polyethylene glenosphere and a metallic humeral inlay. The glenoid component features a two-peg design, a superior angular stable locking screw, and lateral and posterior fixation screws. The humeral stem has different designs for both cemented and uncemented fixation.

For radiographic imaging, we followed a standard protocol described previously.^18^ We assessed scapular notching according to the Nerot-Sirveaux scapular notching classification,^32^^,^^34^ which was adapted as described previously.^18^

Clinical assessment was performed using the modified Constant-Murley score,^6^ the American Shoulder and Elbow Surgeons score,^30^ and range of motion (ROM) for active forward flexion, abduction, as well as internal and external rotation at 90°. We assessed patient pain and satisfaction using the visual analog scale. All complications were systematically recorded, and prosthesis survival rates for any reason of re-revision (revision after failure of revision RTSA) were calculated.

### Statistical analysis

We presented descriptive statistical figures for continuous variables using means, medians, and ranges. Categorical data were presented as frequencies and percentages, and group comparisons for continuous variables were evaluated using the nonparametric Wilcoxon two-sample test. Prosthesis survival was analyzed using the Kaplan-Meier method, with patients censored at death or lost to follow-up. Confidence intervals were calculated using the LOGLOG transformation. We defined final follow-up as the last date an evaluation was made. Lastly, level of significance was set at *P* < .05 (2-sided).

We performed statistical analyses with the Statistical Analysis System (SAS), version 9.4 (SAS Institute Inc., Cary, NC, USA).

## Results

A total of 45 patients (45 shoulders; 28 women and 17 men) underwent revision RTSA for failed primary anatomic shoulder arthroplasty (30 patients) and failed primary RTSA (15 patients). The mean age of the patients was 70.0 years (range, 49.8-86.8 years), and patients who received revision RTSA from primary RTSA were significantly older than those who underwent revision RTSA from anatomic shoulder arthroplasty (mean 74.2 years vs. mean 67.9 years, *P* = .015).

Reasons for primary shoulder arthroplasty included fracture (60%), osteoarthritis (18%), other reasons (9%), cuff tear arthropathy (7%), and post-traumatic arthritis (7%) and were similar between the anatomic shoulder arthroplasty and RTSA groups (*P* = .079). Reasons for revision RTSA, irrespective of the type of index procedure, included infection (22%), secondary tuberosity dislocation or malunion (22%), humeral component malpositioning or loosening (13%), dislocation or instability (13%), glenoid component loosening (11%), rotator cuff disorder (9%), glenoid erosion after HA (4%), and periprosthetic fracture on the humeral side (4%) and were significantly different between the two groups (*P* = .019). Reasons for revision RTSA stratified by the primary procedure are shown in Table I.

**Table I:** Reasons for revision RTSA.

Reasons for revision RTSA	Revision from anatomic shoulder arthroplasty to RTSA n (%)	Revision from RTSA to RTSA n (%)
Glenoid erosion after hemiprosthesis	2 (7)	0 (0)
Glenoid component loosening	2 (7)	3 (20)
Humeral component malpositioning or loosening	2 (7)	4 (27)
Infection	6 (20)	4 (27)
Dislocation or instability	4 (13)	2 (13)
Periprosthetic fracture on the humeral side	0 (0)	2 (13)
Rotator cuff disorder	4 (13)	0 (0)
Secondary tuberosity dislocation or malunion	10 (33)	0 (0)

*RTSA*, reverse total shoulder arthroplasty; *n*, number of shoulders.

**Figure 1:**
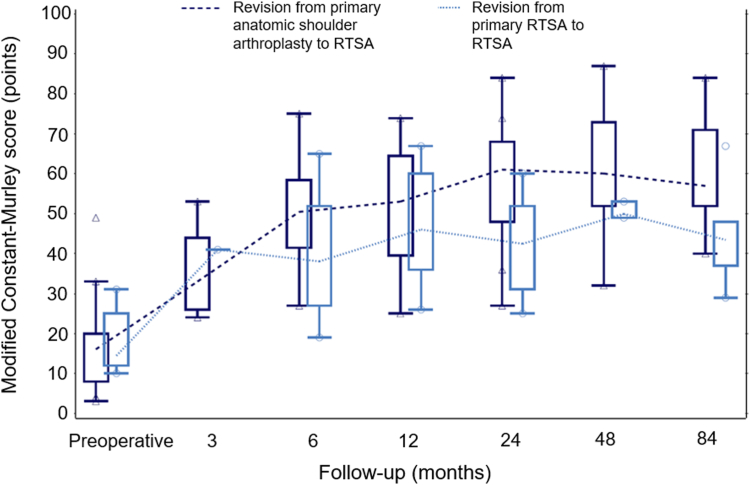
Box plot showing mean modified Constant-Murley scores over the entire follow-up period, stratified by the primary procedure. *RTSA*, reverse total shoulder arthroplasty.

**Figure 2:**
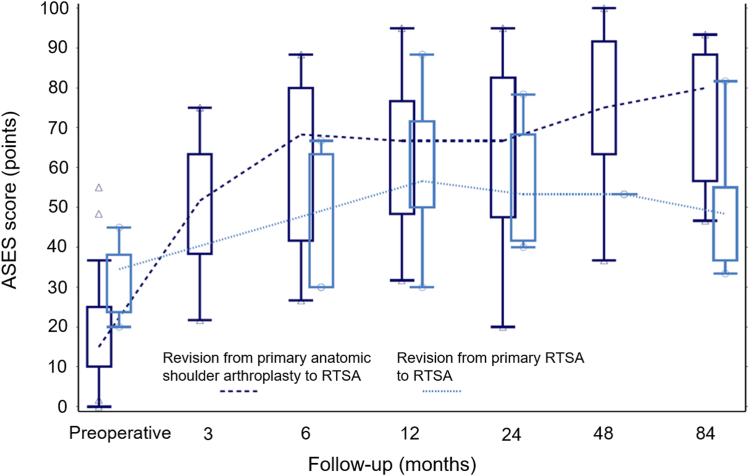
Box plot showing mean American Shoulder and Elbow Surgeons scores over the entire follow-up period, stratified by the primary procedure. *RTSA*, reverse total shoulder arthroplasty; *ASES*, American Shoulder and Elbow Surgeons.

We examined 36 patients clinically and radiographically at a median follow-up of 101.6 months (range, 19.0-170.0 months); 9 patients were excluded from this analysis due to re-revision. At the final follow-up examination, patients undergoing revision RTSA from primary anatomic shoulder arthroplasty showed significantly higher mean modified constant (*P* = .007) and American Shoulder and Elbow Surgeons (*P* = .038) scores than those undergoing revision RTSA from primary RTSA (Table II). Additionally, both of these scores remained inferior in the latter group over the entire study period (Figs. 1 and 2). All active ROM outcomes were similar between the two groups (*P* > .05), except for abduction, which was significantly higher in patients undergoing revision RTSA from anatomic shoulder arthroplasty (*P* = .032). In addition, visual analog scale for pain and satisfaction improved from preoperative values and was similar in the two groups at the final follow-up examination (*P* > .05). Radiographic scapular notching data were available from 32 shoulders (26 from the primary anatomic shoulder arthroplasty to revision RTSA group, 6 from the primary RTSA to revision RTSA group), and the rate of scapular notching was similar between the two groups (15% vs. 17%, *P* = 1.000).

**Table II:** Clinical outcomes at final follow-up.

Clinical outcomes	Revision from anatomic shoulder arthroplasty to RTSA		Revision from RTSA to RTSA		*P* value
	n	Mean (range)	n	Mean (range)	
Modified CS (points)	26	57.4 (20.0-84.0)	9	39.1 (19.0-67.0)	**.007**
ASES score (points)	26	75.3 (35.0-100.0)	7	57.4 (36.7-81.7)	**.038**
Forward flexion (°)	26	116.2 (40.0-180.0)	9	97.2 (30.0-170.0)	.225
Abduction (°)	26	106.5 (25.0-170.0)	9	76.7 (20.0-120.0)	**.032**
External rotation (°)∗	27	26.7 (0.0-80.0)	9	23.3 (0.0-70.0)	.809
Internal rotation (°)∗	13	32.7 (10.0-75.0)	5	32.0 (10.0-50.0)	.705
VAS for pain	26	1.2 (1.0-6.0)	7	1.7 (1.0-5.0)	.710
VAS for satisfaction	26	8.0 (3.0-10.0)	7	5.9 (3.0-10.0)	.072

*CS*, Constant-Murley score; *ASES*, American Shoulder and Elbow Surgeons; *VAS*, visual analog scale; *n*, number of shoulders; *RTSA*, reverse total shoulder arthroplasty.

*P* values from the Wilcoxon two-sample test; bold *P* values refer to significant differences between groups.

∗ External and internal rotation at 90° of abduction.

Complications were recorded in all 45 patients; 8 patients died over the follow-up period. We observed postoperative complications in 14 patients including dislocation, late infection (at >4 weeks postoperatively), periprosthetic fracture of the humerus, and atrophy of the deltoid muscle. Although the overall complication rate was comparable between both groups (*P* = .111), the rates of late infection, dislocation, and periprosthetic fracture were higher in patients who underwent revision RTSA from primary RTSA (Table III). Re-revision was required in nine patients, and the re-revision rate was significantly higher in patients who underwent revision RTSA from primary RTSA than in those who underwent revision RTSA from primary anatomic shoulder arthroplasty (*P* = .018) (Table III). The main reasons for re-revision overall included late infection (44%), periprosthetic fracture (33%), dislocation (11%), and atrophy of the deltoid muscle (11%).

**Table III:** Complications and re-revisions.

Complications and re-revisions	Revision from anatomic shoulder arthroplasty to RTSA n (%)	Revision from RTSA to RTSA n (%)	*P* value
Total complications	7 (23)	7 (47)	.111
*Atrophy of the deltoid muscle*	*1 (3)*	*0 (0)*	
*Late infection*	*1 (3)*	*3 (20)*	
*Dislocation*	*3 (10)*	*2 (13)*	
*Periprosthetic fracture*	*2 (7)*	*2 (13)*	
Re-revisions	3 (10)	6 (40)	.018

*RTSA*, reverse total shoulder arthroplasty; *n*, number of shoulders.

*P* values from the chi-square test.

**Figure 3:**
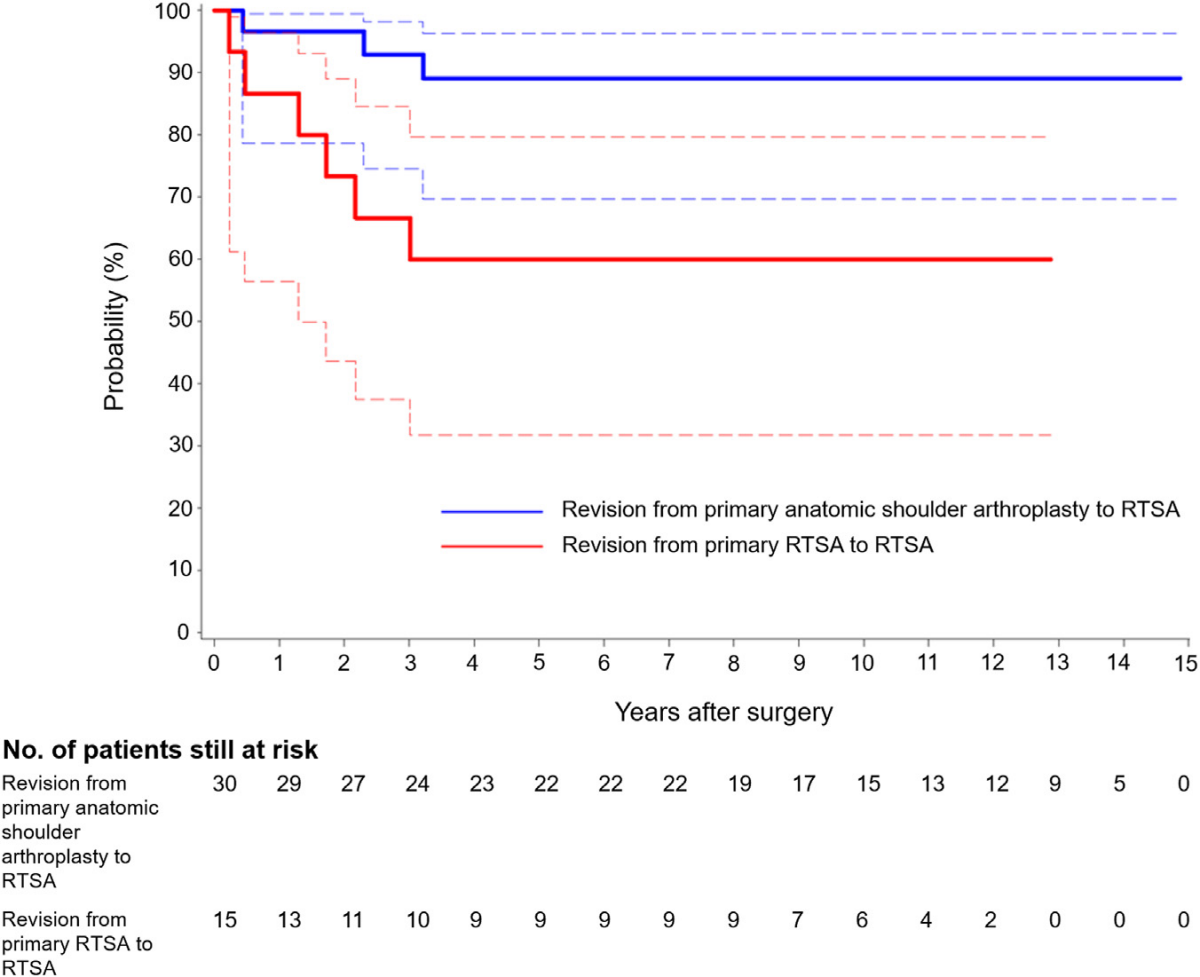
Kaplan-Meier prosthesis survival curves for any reason of re-revision, stratified by the primary procedure. Dashed lines represent *upper* and *lower* limits of 95% confidence intervals. *RTSA*, reverse total shoulder arthroplasty.

Prosthesis survival rates were evaluated in all 45 patients. Overall, prosthesis survival at 10 years was significantly lower in patients who underwent revision RTSA from primary RTSA than those who underwent revision RTSA from primary anatomic shoulder arthroplasty: 60.0% (95% confidence interval, 31.7%-79.6%) vs. 89.0% (95% confidence interval, 69.7%-96.3%) for any reason of re-revision (*P* = .015). Additionally, prosthesis survival remained lower in patients in the former group over the entire follow-up period (Fig. 3).

## Discussion

In this study, we compared clinical outcomes and prosthesis survival rates between patients who underwent revision RTSA with an inverted-bearing prosthesis system for failed primary anatomic shoulder arthroplasty and failed primary RTSA. Our results showed that although revision RTSA successfully reduced pain in all patients, those with anatomic shoulder arthroplasty as the primary procedure experienced better long-term clinical outcomes and prosthesis survival rates after revision RTSA than patients with RTSA as the primary procedure, rejecting our hypothesis. Thus, primary anatomic shoulder arthroplasty could still be considered a good choice, especially for younger patients who are likely to undergo revision surgery in their future lives. In fact, the authors of a recent study reported a lifetime risk of revision surgery of 35.8% after anatomic shoulder arthroplasty in patients aged 50 years or younger.^37^ This must be taken into consideration when performing primary anatomic shoulder arthroplasty, and expanding the indications of primary RTSA without hesitation to all degenerative indications must be viewed critically.

To our knowledge, this is the first study reporting such long-term results of revision RTSA stratified by the primary procedure. A previous study compared clinical outcomes between patients who also underwent RTSA from primary TSA and primary RTSA but found contrasting results: they reported no significant differences in clinical scores, shoulder pain, ROM outcomes, complication rates, and re-revision rates between the two groups (*P* > .05).^14^ We suspect several possible reasons for this difference. First, the age of the patients in their study groups was comparable (*P* = .286), while patients in our study who underwent revision RTSA from failed primary RTSA were significantly older than those who underwent the same procedure from failed primary anatomic shoulder arthroplasty (*P* = .015), explaining the inferior clinical results observed in the former group. Second, the reasons for primary arthroplasty in their study population and ours were fundamentally different: degenerative diseases and rotator cuff arthropathy were the main reasons for primary anatomic shoulder arthroplasty and RTSA in their study, while most of our cases (60%) underwent primary shoulder arthroplasty for fracture. In fact, previously published studies have shown that patients initially treated for proximal humeral fractures have a lower subjective outcome and a higher complication rate after revision RTSA than patients initially treated for osteoarthritis.^9^^,^^17^^,^^22^^,^^23^^,^^28^ Third, the authors excluded all cases of infection from their study population, while our study included 22% of infection cases that underwent revision RTSA. Patients revised for infection often involve chronic infection and are challenging to revise successfully; thus, revision for infection results in worse clinical outcomes compared with revision for other etiologies.^20^ Finally, our results are reported at a longer follow-up period than theirs (mean follow-up of up to 54 months), which could have contributed to the differences.

Another study found no significant difference in re-revision rates 5 years after revision RTSA performed for failed primary anatomic shoulder arthroplasty or RTSA (37.9% vs. 45.5%, *P* = .773).^26^ They also found age and preoperative diagnosis did not affect the re-revision rates after revision RTSA.^26^ However, it must be noted that the follow-up period in this study was shorter than in ours, which could have affected re-revision rates.

Several studies have found improved clinical outcomes after revision RTSA for failed primary shoulder arthroplasty; however, they remain inferior to those after a primary RTSA procedure.^2^^,^^3^^,^^7^^,^^27^^,^^28^^,^^31^ Similarly, revision of failed shoulder arthroplasty to RTSA has shown better clinical outcomes and lower complication rates than revision to another anatomic shoulder arthroplasty.^24^^,^^29^ Taken together, these results indicate that revision RTSA remains a viable treatment option for failed primary shoulder arthroplasty.

Although the overall complication rate was comparable between both groups (*P* = .111), we observed higher rates of late infection, dislocation, and periprosthetic fracture in patients who were revised from primary RTSA than those who were revised from primary anatomic shoulder arthroplasty. The re-revision rate of revision RTSA from primary RTSA in our study, on the other hand, was higher than the re-revision rate of 24% reported previously at 8 years.^12^ However, it should be noted that this study excluded any cases with infection for revision RTSA, which could have affected the re-revision outcomes. The higher re-revision rate in patients revised to RTSA from primary RTSA in our study is most likely due to the increased infection susceptibility of RTSA patients and possibly due to deltoid atrophy (decreased deltoid function). In fact, RTSA results in higher rates of infection than TSA due to formation of hematomas and the dead space caused by the lack of a rotator cuff and distalization of the implant.^10^ Since we were unable to retrieve any other long-term follow-up studies on re-revision rates after revision RTSA, any further analysis of re-revision rates is limited due to the small sample size of our study.

The main strengths of our study are its multicenter setup, long-term follow-up, and inclusion of cases covering the full spectrum of indications from degenerative conditions to trauma. However, we acknowledge some limitations. First, the sample size of the revision RTSA from a failed primary RTSA group was small. However, it is not unusual for studies on revision RTSA outcomes to have smaller patient populations because most of these patients are rather old, with mean ages at implantation reaching the 70s or above. Nevertheless, with the increasing use of RTSA as a primary procedure and its use in younger patients, future studies will benefit from larger sample sizes. Second, patients revised from failed primary RTSA were significantly older than those revised from primary anatomic shoulder arthroplasty, which may have contributed to the differences in clinical outcomes, re-revision rates, and types of complications between the two groups. This has to do with the fact that in previous years, only older patients were operated on with RTSA, and the indication for RTSA has been expanded to younger patients only recently. However, some authors did not find age to affect re-revision rates after revision RTSA.^26^ Third, we used an inverted-bearing prosthesis system for revision RTSA, for which long-term clinical data are not yet available. Therefore, the results of this study may not be generalized to patients undergoing revision RTSA with conventional reverse prosthesis systems. In addition, it is impossible to isolate which index procedure (anatomical TSA or HA) and which indication contributed most favorably to the clinical outcomes. Last, the heterogeneity in our patient collective with various reasons for primary shoulder arthroplasty and significantly different reasons for revision RTSA could have led to results different from those of other published studies. Future studies on revision RTSA, including patients with more homogeneous indications for the primary procedure and similar reasons for revision procedure, will be needed to confirm our results.

## Conclusions

We found better long-term clinical scores, abduction, and prosthesis survival rates after revision RTSA with an inverted-bearing prosthesis system in patients with primary anatomic shoulder arthroplasty than those with primary RTSA. Nevertheless, patients in both groups experienced a similar reduction in pain postoperatively, and the overall complication rates were comparable in both groups. Our results suggest that anatomic shoulder arthroplasty could be an attractive option for primary arthroplasty, especially in younger patients who are likely to undergo revision surgery in their future lives. However, future studies with larger sample sizes are needed to confirm these results.

## Acknowledgments

The authors thank Dr. Dominik Pfluger at numerics data GmbH for statistical analysis, Mathys Ltd for partially funding this study, and Medical Minds GmbH for providing medical writing and editorial support.

## Disclaimers:

Funding: The study was partially funded by Mathys Ltd Bettlach. Funds sponsored statistical analysis through an independent consultant, medical advisor contracts and travel expenses for Falk Reuther, Ulrich Irlenbusch, Georges Kohut, Thierry Joudet, and Max J. Kääb, as well as medical writing and editorial support from a medical writing agency. Mathys Ltd Bettlach was not involved in the design or execution of the study, the analysis or interpretation of the data, or the decision to submit the results.

Conflicts of interest: The authors, their immediate families, and any research foundation with which they are affiliated have not received any financial payments or other benefits from any commercial entity related to the subject of this article.
